# The link between employee attitudes and employee effectiveness: Data matrix of meta-analytic estimates based on 1161 unique correlations

**DOI:** 10.1016/j.dib.2016.08.002

**Published:** 2016-08-06

**Authors:** Michael M. Mackay

**Affiliations:** The University of Memphis, United States

**Keywords:** Meta-analysis, Job attitudes, Job performance, Employee, Engagement, Employee effectiveness

## Abstract

This article offers a correlation matrix of meta-analytic estimates between various employee job attitudes (i.e., Employee engagement, job satisfaction, job involvement, and organizational commitment) and indicators of employee effectiveness (i.e., Focal performance, contextual performance, turnover intention, and absenteeism). The meta-analytic correlations in the matrix are based on over 1100 individual studies representing over 340,000 employees. Data was collected worldwide via employee self-report surveys. Structural path analyses based on the matrix, and the interpretation of the data, can be found in “*Investigating the incremental validity of employee engagement in the prediction of employee effectiveness: a meta-analytic path analysis*” (Mackay et al., 2016) [1].

**Specifications Table**

**Table:** 

Subject area	*Psychology*
More specific subject area	*Industrial-Organizational Psychology*
Type of data	*Table*
How data was acquired	*Surveys of employees, coworkers, and supervisors*
Data format	*Analyzed*
Experimental factors	*Data were collected via self-report questionnaires of current employees*
Experimental features	*Correlations disattenuated for unreliability and, when possible, non-common source estimates used*
Data source location	*Worldwide, with majority from U.S. and European sources*
Data accessibility	*Data is within this article*

**Value of the data**

• Analyses can be performed in the future to compute updated meta-analytic estimates as new research becomes available.
• Other variables (e.g., workplace deviance, lateness, counterproductive work behavior) can be added to the meta-matrix to make it more comprehensive.
• Issues relating to the discriminant, convergent, and incremental validity of various job attitudes can be explored.

## 1 Data

The correlation matrix (see Table 1 in the online version of this article) contains meta-analytic estimates between job attitudes (Employee engagement, job satisfaction, job involvement, organizational commitment) and indicators of employee effectiveness (Focal performance, contextual performance, turnover intention, absenteeism). The meta-matrix is based on 1161 previously published correlations. The meta-analytic estimates are disattenuated for unreliability in both the predictor and the criterion (except absenteeism, see below). Estimates relating to focal and contextual performance come from non-common source estimates only, thus avoiding the possibility of common-method bias. No corrections for range restriction were conducted due to unavailability of the data. Lastly, estimates relating to absenteeism are based on objective measures and considered perfectly reliable.

## 2 Experimental design, materials and methods

### 2.1 Identification of studies relating to employee engagement

Of the 28 cells in the correlational matrix, four cells represent newly computed meta-analytic estimates between employee engagement (EE) and various indicators of employee effectiveness. To identify studies that would be used to compute these four meta-analytic estimates, a search was performed using PsycINFO, Business Source Premier, ABI/INFORM and Web of Science databases for the years 1990–2015. Common synonyms were used as subject term/keywords to ensure a thorough search (e.g., along with “employee engagement,” the terms “work engagement” and “job engagement” were also used). To be included, a study had to present original data, provide the sample size, and report a correlation coefficient between EE and one of the four employee effectiveness indicators.

### 2.2 Meta-analytic procedures relating to employee engagement

For the four cells of the meta-matrix that show correlations between EE and indicators of employee effectiveness, Hunter and Schmidt׳s [2] meta-analytic procedures were used to make corrections for sampling error and unreliability in predictor and criterion measures. For the few studies in which Cronbach׳s alpha was not reported (<5%), the mean reliability of the instrument across all other studies was computed and used as a proxy. No corrections were made for range restriction due to unavailability of this data. Objective measures of absenteeism were considered perfectly reliable and not corrected. Lastly, all measures of focal and contextual job performance were either supervisor- or coworker-rated (i.e., they were not based on self ratings).

### 2.3 Identification of studies for the remainder of meta-matrix

As mentioned above, four of the 28 cells of the correlational matrix represent newly computed meta-analytic estimates between employee engagement (EE) and various indicators of employee effectiveness. The remaining 24 cells of the correlational matrix are populated with estimates taken from existing meta-analyses. The criteria for inclusion for these estimates were that they came from the most comprehensive meta-analyses to date (i.e., were derived from the largest number of original studies), the correlations were corrected for measurement unreliability, and estimates relating to focal and contextual job performance were based on non-common source estimates only.

Specifically, data from the following meta-analyses were to populate the corresponding cells in the meta-matrix (see [1] for details):

• Mackay et al. [1]: employee engagement and focal performance, employee engagement and contextual performance, employee engagement and turnover intention, employee engagement and absenteeism.
• Meyer et al. [3]: job satisfaction and organizational commitment, contextual performance and organizational commitment, job involvement and organizational commitment.
• Brown [4]: job satisfaction and job involvement, job involvement and absenteeism.
• Judge et al. [5]: job satisfaction and focal performance.
• Ilies et al. [6]: job satisfaction and contextual performance.
• Griffeth et al. [7]: job satisfaction and turnover intention, organizational commitment and turnover intention, focal performance and turnover intention.
• Hackett [8]: job satisfaction and absenteeism.
• Riketta [9]: organizational commitment and focal performance.
• Harrison et al. [10]: organizational commitment and absenteeism, focal performance and contextual performance, contextual performance and turnover intention, contextual performance and absenteeism.
• Joseph et al. [11]: employee engagement and job satisfaction, employee engagement and organizational commitment, employee engagement and job involvement, job involvement and focal performance, job involvement and contextual performance, job involvement and turnover intention.
• Bycio [12]: focal performance and absenteeism.
• Mitra (1992) [13]: turnover intention and absenteeism.
