# Supplementary material for: The link between employee attitudes and employee effectiveness: Data matrix of meta-analytic estimates based on 1161 unique correlations
Source: Data Brief. 2016 Aug 6;8:1391–4. doi: 10.1016/j.dib.2016.08.002 (PMC4993852; doi:10.1016/j.dib.2016.08.002)
Supplement: Supplementary file 1 — Supplementary material [file mmc1.docx]

[No Conflict of Interest form is provided on the Elsevier website]

There are no conflicts of interest. All data is previously published in academic journals and publicly available.
